# 5,5′-[(2,4-Dichloro­phen­yl)methyl­ene]bis­(2,2-dimethyl-1,3-dioxane-4,6-dione)

**DOI:** 10.1107/S1600536811025384

**Published:** 2011-07-02

**Authors:** Wu-Lan Zeng

**Affiliations:** a MicroScale Science Institute, Department of Chemistry and Chemical Engineering, Weifang University, Weifang 261061, People’s Republic of China

## Abstract

The title compound, C_19_H_18_Cl_2_O_8_, was prepared by the reaction of 2,2-dimethyl-1,3-dioxane-4,6-dione and 2,4-dichloro­benzaldehyde in ethanol. The two 1,3-dioxane rings exhibit boat conformations. In the crystal, mol­ecules are linked by weak inter­molecular C—H⋯O and C—H⋯Cl hydrogen bonds, forming chains parallel to the *a* axis.

## Related literature

For related structures, see: Zeng (2010 ▶, 2011 ▶).
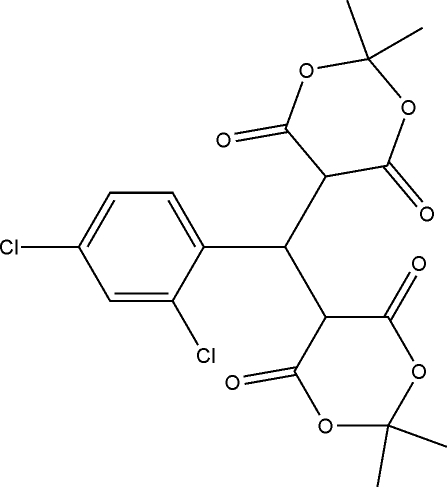


## Experimental

### 

#### Crystal data

C_19_H_18_Cl_2_O_8_
*M* *_r_* = 445.23
Monoclinic, 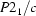

*a* = 7.9522 (6) Å
*b* = 11.5145 (11) Å
*c* = 22.0939 (19) Å
β = 100.201 (1)°
*V* = 1991.1 (3) Å^3^
*Z* = 4
Mo *K*α radiation
μ = 0.37 mm^−1^
*T* = 298 K
0.40 × 0.34 × 0.28 mm

#### Data collection

Bruker SMART CCD area-detector diffractometer
Absorption correction: multi-scan (*SADABS*; Sheldrick, 1996) ▶ *T*_min_ = 0.866, *T*_max_ = 0.903
9830 measured reflections
3519 independent reflections
1978 reflections with *I* > 2σ(*I*)
*R*_int_ = 0.039

#### Refinement

*R*[*F* ^2^ > 2σ(*F* ^2^)] = 0.045
*wR*(*F* ^2^) = 0.127
*S* = 1.03
3519 reflections
266 parameters
H-atom parameters constrained
Δρ_max_ = 0.33 e Å^−3^
Δρ_min_ = −0.28 e Å^−3^

### 

Data collection: *SMART* (Bruker, 1997 ▶); cell refinement: *SAINT* (Bruker, 1997 ▶); data reduction: *SAINT*; program(s) used to solve structure: *SHELXS97* (Sheldrick, 2008 ▶); program(s) used to refine structure: *SHELXL97* (Sheldrick, 2008 ▶); molecular graphics: *SHELXTL* (Sheldrick, 2008 ▶); software used to prepare material for publication: *SHELXTL*.

## Supplementary Material

Crystal structure: contains datablock(s) global, I. DOI: 10.1107/S1600536811025384/rz2616sup1.cif

Structure factors: contains datablock(s) I. DOI: 10.1107/S1600536811025384/rz2616Isup2.hkl

Supplementary material file. DOI: 10.1107/S1600536811025384/rz2616Isup3.cml

Additional supplementary materials:  crystallographic information; 3D view; checkCIF report

## Figures and Tables

**Table 1:** Hydrogen-bond geometry (Å, °)

*D*—H⋯*A*	*D*—H	H⋯*A*	*D*⋯*A*	*D*—H⋯*A*
C8—H8⋯O4^i^	0.98	2.32	3.220 (4)	151
C11—H11*B*⋯Cl1^ii^	0.96	2.75	3.387 (4)	125
C11—H11*C*⋯O4^i^	0.96	2.43	3.323 (4)	155

Symmetry codes: (i) 
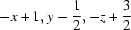
; (ii) 
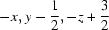
.

**Fig. 1.**
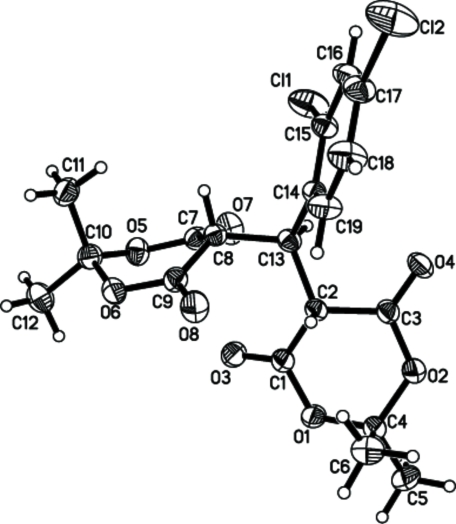
The molecular structure of the title compound, drawn with 30% probability ellipsoids and spheres of arbritrary size for the H atoms.

## supplementary crystallographic information

### Comment

In previous papers, the crystal structure of 5-(4-hydroxybenzylidene)-2,2-dimethyl-1,3-dioxane-4,6-dione (Zeng, 2010) and 2,2-dimethyl-5-[(5-methylfuran-2-yl)methylidene]-1,3-dioxane-4,6-dione (Zeng, 2011) have been reported. As part of this ongoing search for new Meldrum's acid compounds, the title compound  has been synthesized and its structure is reported here.

In the title compound (Fig. 1), bond lengths and angles fall in the usual ranges. The two 1,3-dioxane rings exhibit boat conformations. In the crystal structure, the molecules interact through weak intermolecular C—H···O and C—H···Cl hydrogen bonds (Table 1) to form chains parallel to the *a* axis.

### Experimental

A mixture of malonic acid (6.24 g, 0.06 mol) and acetic anhydride (9 ml) in concentrated sulfuric acid (0.25 ml) was stirred with water at 303 K, After dissolving, propan-2-one (3.48 g, 0.06 mol) was added dropwise into solution for 1 h. The reaction was allowed to proceed for 2 h. The mixture was cooled and filtered, and then an ethanol solution of 2,4-dichlorobenzaldehyde (10.44 g,0.06 mol) was added. The solution was then filtered and concentrated. Single crystals were obtained by evaporation of an petroleum ether/acetone (1:1 *v*/*v*) solution at room temperature over a period of several days.

### Refinement

The H atoms were placed in calculated positions (C—H = 0.93–0.98 Å), and refined as riding with *U*_iso_(H) = 1.2*U*_eq_(C) or 1.5U_eq_(C) for methyl H atoms.

### Crystal data

**Table:**

C_19_H_18_Cl_2_O_8_	*F*(000) = 920
*M**_r_* = 445.23	*D*_x_ = 1.485 Mg m^−^^3^
Monoclinic, *P*2_1_/*c*	Mo *K*α radiation, λ = 0.71073 Å
Hall symbol: -P 2ybc	Cell parameters from 2055 reflections
*a* = 7.9522 (6) Å	θ = 2.6–22.4°
*b* = 11.5145 (11) Å	µ = 0.37 mm^−^^1^
*c* = 22.0939 (19) Å	*T* = 298 K
β = 100.201 (1)°	Block, colourless
*V* = 1991.1 (3) Å^3^	0.40 × 0.34 × 0.28 mm
*Z* = 4	

### Data collection

**Table:**

Bruker SMART CCD area-detector diffractometer	3519 independent reflections
Radiation source: fine-focus sealed tube	1978 reflections with *I* > 2σ(*I*)
graphite	*R*_int_ = 0.039
phi and ω scans	θ_max_ = 25.0°, θ_min_ = 2.6°
Absorption correction: multi-scan (*SADABS*; Sheldrick, 1996)	*h* = −8→9
*T*_min_ = 0.866, *T*_max_ = 0.903	*k* = −13→13
9830 measured reflections	*l* = −26→16

### Refinement

**Table:**

Refinement on *F*^2^	Primary atom site location: structure-invariant direct methods
Least-squares matrix: full	Secondary atom site location: difference Fourier map
*R*[*F*^2^ > 2σ(*F*^2^)] = 0.045	Hydrogen site location: inferred from neighbouring sites
*wR*(*F*^2^) = 0.127	H-atom parameters constrained
*S* = 1.03	*w* = 1/[σ^2^(*F*_o_^2^) + (0.0473*P*)^2^ + 0.9163*P*] where *P* = (*F*_o_^2^ + 2*F*_c_^2^)/3
3519 reflections	(Δ/σ)_max_ < 0.001
266 parameters	Δρ_max_ = 0.33 e Å^−^^3^
0 restraints	Δρ_min_ = −0.28 e Å^−^^3^

### Special details

**Table:**

Geometry. All e.s.d.'s (except the e.s.d. in the dihedral angle between two l.s. planes) are estimated using the full covariance matrix. The cell e.s.d.'s are taken into account individually in the estimation of e.s.d.'s in distances, angles and torsion angles; correlations between e.s.d.'s in cell parameters are only used when they are defined by crystal symmetry. An approximate (isotropic) treatment of cell e.s.d.'s is used for estimating e.s.d.'s involving l.s. planes.
Refinement. Refinement of *F*^2^ against ALL reflections. The weighted *R*-factor *wR* and goodness of fit *S* are based on *F*^2^, conventional *R*-factors *R* are based on *F*, with *F* set to zero for negative *F*^2^. The threshold expression of *F*^2^ > σ(*F*^2^) is used only for calculating *R*-factors(gt) *etc*. and is not relevant to the choice of reflections for refinement. *R*-factors based on *F*^2^ are statistically about twice as large as those based on *F*, and *R*- factors based on ALL data will be even larger.

### Fractional atomic coordinates and isotropic or equivalent isotropic displacement parameters (Å^2^)

**Table:**

	*x*	*y*	*z*	*U*_iso_*/*U*_eq_	
Cl1	0.34140 (13)	0.58466 (10)	0.82975 (4)	0.0791 (4)	
Cl2	0.94290 (17)	0.39837 (13)	0.91959 (5)	0.1097 (5)	
O1	0.4381 (3)	0.67539 (19)	0.54288 (9)	0.0534 (6)	
O2	0.6398 (3)	0.77237 (18)	0.61607 (10)	0.0519 (6)	
O3	0.2390 (3)	0.5720 (2)	0.57392 (10)	0.0633 (7)	
O4	0.6173 (3)	0.75628 (19)	0.71323 (10)	0.0563 (7)	
O5	−0.0131 (3)	0.43266 (19)	0.63977 (10)	0.0509 (6)	
O6	0.1923 (3)	0.30698 (19)	0.61015 (10)	0.0530 (6)	
O7	0.0621 (3)	0.5985 (2)	0.68504 (11)	0.0633 (7)	
O8	0.4641 (3)	0.3549 (2)	0.62659 (11)	0.0576 (6)	
C1	0.3785 (5)	0.6132 (3)	0.58613 (14)	0.0464 (8)	
C2	0.5000 (4)	0.6011 (2)	0.64670 (13)	0.0393 (8)	
H2	0.5877	0.5452	0.6399	0.047*	
C3	0.5907 (4)	0.7152 (3)	0.66308 (15)	0.0422 (8)	
C4	0.6071 (4)	0.7239 (3)	0.55467 (14)	0.0483 (9)	
C5	0.6000 (5)	0.8257 (3)	0.51133 (15)	0.0637 (11)	
H5A	0.5794	0.7982	0.4697	0.096*	
H5B	0.7067	0.8667	0.5193	0.096*	
H5C	0.5093	0.8770	0.5174	0.096*	
C6	0.7397 (5)	0.6361 (3)	0.54565 (16)	0.0649 (10)	
H6A	0.7325	0.5703	0.5718	0.097*	
H6B	0.8512	0.6703	0.5559	0.097*	
H6C	0.7204	0.6113	0.5035	0.097*	
C7	0.1059 (4)	0.5050 (3)	0.67033 (14)	0.0453 (8)	
C8	0.2861 (4)	0.4576 (3)	0.68544 (13)	0.0375 (7)	
H8	0.2904	0.4136	0.7237	0.045*	
C9	0.3256 (5)	0.3709 (3)	0.63918 (14)	0.0433 (8)	
C10	0.0272 (4)	0.3142 (3)	0.62803 (15)	0.0486 (8)	
C11	0.0244 (5)	0.2407 (3)	0.68364 (16)	0.0604 (10)	
H11A	0.0455	0.1612	0.6742	0.091*	
H11B	−0.0853	0.2469	0.6957	0.091*	
H11C	0.1114	0.2669	0.7166	0.091*	
C12	−0.1003 (5)	0.2776 (4)	0.57242 (16)	0.0731 (12)	
H12A	−0.0942	0.3298	0.5390	0.110*	
H12B	−0.2133	0.2795	0.5821	0.110*	
H12C	−0.0746	0.2002	0.5608	0.110*	
C13	0.4220 (4)	0.5546 (3)	0.70107 (13)	0.0385 (7)	
H13	0.3633	0.6205	0.7162	0.046*	
C14	0.5585 (4)	0.5159 (2)	0.75482 (13)	0.0360 (7)	
C15	0.5303 (4)	0.5281 (3)	0.81477 (14)	0.0432 (8)	
C16	0.6490 (5)	0.4927 (3)	0.86523 (14)	0.0524 (9)	
H16	0.6284	0.5041	0.9049	0.063*	
C17	0.7951 (5)	0.4412 (3)	0.85586 (16)	0.0552 (9)	
C18	0.8268 (5)	0.4243 (3)	0.79810 (17)	0.0646 (11)	
H18	0.9268	0.3878	0.7921	0.078*	
C19	0.7089 (4)	0.4620 (3)	0.74818 (15)	0.0538 (9)	
H19	0.7321	0.4505	0.7088	0.065*	

### Atomic displacement parameters (Å^2^)

**Table:**

	*U*^11^	*U*^22^	*U*^33^	*U*^12^	*U*^13^	*U*^23^
Cl1	0.0685 (7)	0.1167 (9)	0.0547 (6)	0.0359 (6)	0.0181 (5)	−0.0067 (6)
Cl2	0.0957 (9)	0.1545 (12)	0.0645 (7)	0.0415 (8)	−0.0254 (6)	0.0215 (7)
O1	0.0564 (15)	0.0646 (15)	0.0350 (13)	−0.0160 (12)	−0.0035 (10)	0.0125 (11)
O2	0.0649 (16)	0.0464 (13)	0.0429 (14)	−0.0127 (11)	0.0053 (11)	−0.0009 (11)
O3	0.0572 (17)	0.0817 (18)	0.0445 (14)	−0.0252 (14)	−0.0084 (11)	0.0136 (12)
O4	0.0752 (18)	0.0515 (14)	0.0378 (14)	−0.0083 (12)	−0.0014 (12)	−0.0084 (11)
O5	0.0375 (14)	0.0526 (14)	0.0589 (15)	−0.0018 (11)	−0.0017 (10)	0.0004 (11)
O6	0.0548 (16)	0.0554 (15)	0.0494 (14)	−0.0127 (12)	0.0112 (11)	−0.0114 (11)
O7	0.0477 (16)	0.0586 (17)	0.0805 (18)	0.0128 (12)	0.0029 (12)	−0.0087 (13)
O8	0.0479 (16)	0.0574 (15)	0.0708 (17)	−0.0008 (12)	0.0200 (13)	−0.0130 (12)
C1	0.051 (2)	0.048 (2)	0.0379 (19)	−0.0090 (17)	0.0009 (16)	0.0024 (15)
C2	0.0433 (19)	0.0407 (18)	0.0308 (17)	−0.0043 (15)	−0.0014 (13)	0.0011 (14)
C3	0.046 (2)	0.0401 (19)	0.038 (2)	−0.0034 (15)	−0.0009 (15)	0.0013 (16)
C4	0.052 (2)	0.053 (2)	0.0368 (19)	−0.0139 (18)	0.0017 (15)	0.0010 (16)
C5	0.076 (3)	0.064 (2)	0.052 (2)	−0.012 (2)	0.0134 (19)	0.0162 (19)
C6	0.070 (3)	0.070 (3)	0.056 (2)	0.005 (2)	0.0158 (19)	0.0018 (19)
C7	0.043 (2)	0.050 (2)	0.0423 (19)	0.0013 (17)	0.0040 (15)	0.0070 (17)
C8	0.0324 (18)	0.0442 (18)	0.0349 (17)	0.0001 (14)	0.0034 (13)	0.0028 (14)
C9	0.046 (2)	0.0400 (19)	0.044 (2)	−0.0019 (16)	0.0068 (16)	0.0028 (15)
C10	0.041 (2)	0.057 (2)	0.047 (2)	−0.0057 (17)	0.0045 (15)	0.0022 (17)
C11	0.057 (2)	0.060 (2)	0.062 (2)	−0.0143 (19)	0.0053 (18)	0.0097 (19)
C12	0.065 (3)	0.088 (3)	0.059 (3)	−0.022 (2)	−0.0093 (19)	−0.006 (2)
C13	0.0388 (19)	0.0397 (18)	0.0350 (17)	0.0031 (14)	0.0011 (13)	0.0013 (14)
C14	0.0363 (18)	0.0382 (17)	0.0325 (17)	−0.0001 (14)	0.0032 (13)	0.0029 (13)
C15	0.0409 (19)	0.050 (2)	0.0384 (19)	0.0044 (15)	0.0064 (14)	−0.0030 (15)
C16	0.061 (2)	0.064 (2)	0.0318 (19)	−0.0010 (19)	0.0061 (16)	−0.0034 (16)
C17	0.050 (2)	0.067 (2)	0.043 (2)	0.0078 (19)	−0.0061 (16)	0.0111 (18)
C18	0.048 (2)	0.091 (3)	0.054 (2)	0.025 (2)	0.0056 (18)	0.007 (2)
C19	0.047 (2)	0.075 (2)	0.0395 (19)	0.0140 (19)	0.0091 (16)	0.0045 (17)

### Geometric parameters (Å, °)

**Table:**

Cl1—C15	1.722 (3)	C6—H6C	0.9600
Cl2—C17	1.738 (3)	C7—C8	1.515 (4)
O1—C1	1.346 (4)	C8—C9	1.501 (4)
O1—C4	1.436 (4)	C8—C13	1.550 (4)
O2—C3	1.345 (4)	C8—H8	0.9800
O2—C4	1.447 (4)	C10—C11	1.495 (4)
O3—C1	1.193 (4)	C10—C12	1.508 (4)
O4—C3	1.189 (3)	C11—H11A	0.9600
O5—C7	1.349 (4)	C11—H11B	0.9600
O5—C10	1.435 (4)	C11—H11C	0.9600
O6—C9	1.355 (4)	C12—H12A	0.9600
O6—C10	1.439 (4)	C12—H12B	0.9600
O7—C7	1.194 (4)	C12—H12C	0.9600
O8—C9	1.197 (4)	C13—C14	1.527 (4)
C1—C2	1.512 (4)	C13—H13	0.9800
C2—C3	1.511 (4)	C14—C19	1.378 (4)
C2—C13	1.542 (4)	C14—C15	1.389 (4)
C2—H2	0.9800	C15—C16	1.389 (4)
C4—C6	1.500 (5)	C16—C17	1.352 (5)
C4—C5	1.508 (4)	C16—H16	0.9300
C5—H5A	0.9600	C17—C18	1.358 (5)
C5—H5B	0.9600	C18—C19	1.384 (4)
C5—H5C	0.9600	C18—H18	0.9300
C6—H6A	0.9600	C19—H19	0.9300
C6—H6B	0.9600		
			
C1—O1—C4	120.9 (2)	O8—C9—C8	124.9 (3)
C3—O2—C4	120.4 (2)	O6—C9—C8	116.3 (3)
C7—O5—C10	121.2 (2)	O5—C10—O6	110.4 (2)
C9—O6—C10	120.9 (3)	O5—C10—C11	110.7 (3)
O3—C1—O1	118.8 (3)	O6—C10—C11	109.9 (3)
O3—C1—C2	125.9 (3)	O5—C10—C12	105.9 (3)
O1—C1—C2	115.3 (3)	O6—C10—C12	105.9 (3)
C3—C2—C1	109.6 (2)	C11—C10—C12	113.9 (3)
C3—C2—C13	110.9 (2)	C10—C11—H11A	109.5
C1—C2—C13	116.0 (3)	C10—C11—H11B	109.5
C3—C2—H2	106.6	H11A—C11—H11B	109.5
C1—C2—H2	106.6	C10—C11—H11C	109.5
C13—C2—H2	106.6	H11A—C11—H11C	109.5
O4—C3—O2	119.8 (3)	H11B—C11—H11C	109.5
O4—C3—C2	124.6 (3)	C10—C12—H12A	109.5
O2—C3—C2	115.6 (3)	C10—C12—H12B	109.5
O1—C4—O2	109.1 (3)	H12A—C12—H12B	109.5
O1—C4—C6	111.8 (3)	C10—C12—H12C	109.5
O2—C4—C6	111.8 (3)	H12A—C12—H12C	109.5
O1—C4—C5	105.0 (3)	H12B—C12—H12C	109.5
O2—C4—C5	106.0 (3)	C14—C13—C2	112.2 (2)
C6—C4—C5	112.8 (3)	C14—C13—C8	109.6 (2)
C4—C5—H5A	109.5	C2—C13—C8	115.9 (2)
C4—C5—H5B	109.5	C14—C13—H13	106.1
H5A—C5—H5B	109.5	C2—C13—H13	106.1
C4—C5—H5C	109.5	C8—C13—H13	106.1
H5A—C5—H5C	109.5	C19—C14—C15	115.9 (3)
H5B—C5—H5C	109.5	C19—C14—C13	124.0 (3)
C4—C6—H6A	109.5	C15—C14—C13	120.0 (3)
C4—C6—H6B	109.5	C16—C15—C14	122.3 (3)
H6A—C6—H6B	109.5	C16—C15—Cl1	116.7 (3)
C4—C6—H6C	109.5	C14—C15—Cl1	121.0 (2)
H6A—C6—H6C	109.5	C17—C16—C15	119.1 (3)
H6B—C6—H6C	109.5	C17—C16—H16	120.5
O7—C7—O5	118.7 (3)	C15—C16—H16	120.5
O7—C7—C8	125.1 (3)	C16—C17—C18	120.9 (3)
O5—C7—C8	116.1 (3)	C16—C17—Cl2	118.4 (3)
C9—C8—C7	113.2 (3)	C18—C17—Cl2	120.6 (3)
C9—C8—C13	114.1 (3)	C17—C18—C19	119.5 (3)
C7—C8—C13	112.6 (3)	C17—C18—H18	120.3
C9—C8—H8	105.3	C19—C18—H18	120.3
C7—C8—H8	105.3	C14—C19—C18	122.3 (3)
C13—C8—H8	105.3	C14—C19—H19	118.9
O8—C9—O6	118.8 (3)	C18—C19—H19	118.9
			
C4—O1—C1—O3	−178.1 (3)	C7—O5—C10—C11	80.4 (3)
C4—O1—C1—C2	1.0 (4)	C7—O5—C10—C12	−155.7 (3)
O3—C1—C2—C3	−140.5 (4)	C9—O6—C10—O5	41.7 (4)
O1—C1—C2—C3	40.4 (4)	C9—O6—C10—C11	−80.6 (3)
O3—C1—C2—C13	−14.1 (5)	C9—O6—C10—C12	155.9 (3)
O1—C1—C2—C13	166.9 (3)	C3—C2—C13—C14	−69.0 (3)
C4—O2—C3—O4	−179.5 (3)	C1—C2—C13—C14	165.2 (3)
C4—O2—C3—C2	−0.7 (4)	C3—C2—C13—C8	164.1 (2)
C1—C2—C3—O4	138.2 (3)	C1—C2—C13—C8	38.3 (4)
C13—C2—C3—O4	8.9 (4)	C9—C8—C13—C14	−89.5 (3)
C1—C2—C3—O2	−40.5 (4)	C7—C8—C13—C14	139.6 (3)
C13—C2—C3—O2	−169.9 (3)	C9—C8—C13—C2	38.8 (4)
C1—O1—C4—O2	−42.1 (4)	C7—C8—C13—C2	−92.1 (3)
C1—O1—C4—C6	82.0 (4)	C2—C13—C14—C19	−36.7 (4)
C1—O1—C4—C5	−155.4 (3)	C8—C13—C14—C19	93.6 (4)
C3—O2—C4—O1	41.9 (4)	C2—C13—C14—C15	147.1 (3)
C3—O2—C4—C6	−82.3 (4)	C8—C13—C14—C15	−82.6 (3)
C3—O2—C4—C5	154.5 (3)	C19—C14—C15—C16	2.5 (5)
C10—O5—C7—O7	−171.7 (3)	C13—C14—C15—C16	179.0 (3)
C10—O5—C7—C8	6.0 (4)	C19—C14—C15—Cl1	−175.5 (3)
O7—C7—C8—C9	−152.1 (3)	C13—C14—C15—Cl1	1.0 (4)
O5—C7—C8—C9	30.4 (4)	C14—C15—C16—C17	−2.0 (5)
O7—C7—C8—C13	−20.8 (4)	Cl1—C15—C16—C17	176.0 (3)
O5—C7—C8—C13	161.7 (2)	C15—C16—C17—C18	0.2 (6)
C10—O6—C9—O8	173.5 (3)	C15—C16—C17—Cl2	179.5 (3)
C10—O6—C9—C8	−6.4 (4)	C16—C17—C18—C19	1.0 (6)
C7—C8—C9—O8	150.0 (3)	Cl2—C17—C18—C19	−178.3 (3)
C13—C8—C9—O8	19.4 (4)	C15—C14—C19—C18	−1.3 (5)
C7—C8—C9—O6	−30.1 (4)	C13—C14—C19—C18	−177.6 (3)
C13—C8—C9—O6	−160.6 (2)	C17—C18—C19—C14	−0.4 (6)
C7—O5—C10—O6	−41.5 (4)		

### Hydrogen-bond geometry (Å, °)

**Table:**

*D*—H···*A*	*D*—H	H···*A*	*D*···*A*	*D*—H···*A*
C8—H8···O4^i^	0.98	2.32	3.220 (4)	151
C11—H11B···Cl1^ii^	0.96	2.75	3.387 (4)	125
C11—H11C···O4^i^	0.96	2.43	3.323 (4)	155

Symmetry codes: (i) −*x*+1, *y*−1/2, −*z*+3/2; (ii) −*x*, *y*−1/2, −*z*+3/2.
